# Infection néonatale invasive à entérovirus associant une myocardite sévère et une méningite

**DOI:** 10.11604/pamj.2014.19.307.3258

**Published:** 2014-11-21

**Authors:** Boulyana Mohamed

**Affiliations:** 1 Service de Néonatologie, Centre Hospitalier de la Région de Saint Omer, France

**Keywords:** Infection invasive à entérovirus, myocardite, méningite, nouveau né, Invasive enteroviral infection, myocarditis, meningitis, newborn

## Abstract

L'infection néonatale invasive à entérovirus (EV) reste rare mais souvent fatale et devrait être prise dans le diagnostic différentiel chez les enfants septiques. Nous rapportons le cas d'un nouveau-né hospitalisé pour un sepsis grave à EV avec une méningite et une myocardite sévère d'évolution favorable après une corticothérapie. Nous présentons une discussion basée sur une revue de la littérature pour aider le clinicien à reconnaitre les facteurs de gravité et à apprécier les nouvelles stratégies diagnostiques telle la PCR (Polymerase Chain Reaction) et l'imagerie par résonance magnétique (IRM) cardiaque. Nous passons également en revue les nouvelles approches thérapeutiques telles les biothérapies, les antiviraux spécifiques et l'Oxygénation par Membrane Extra-Corporelle (ECMO).

## Introduction

L'infection néonatale invasive à EV reste rare mais elle peut être rapidement fatale surtout en cas de myocardite où la mortalité atteint 44% à 100% [1, 2]. Nous rapportons le cas d'un nouveau-né hospitalisé pour un sepsis grave à EV avec une méningite et une myocardite sévère d'évolution favorable après une corticothérapie. Nous présentons une discussion basée sur une revue de la littérature pour aider le clinicien à apprécier les nouvelles stratégies diagnostiques et thérapeutiques.

## Patient et observation

En été 2009, un nouveau-né de 8 jours sans antécédents pathologiques en dehors et d'un épisode de virose maternelle une semaine avant la naissance et d'une gastro-entérite chez le nouveau né la veille de son hospitalisation. Il a été hospitalisé pour un sepsis sévère avec une hypothermie à 34°C et une insuffisance respiratoire et circulatoire. A l'admission, la radiographie thoracique, l'analyse des urines, la CRP et la procalcitonine étaient normales. Le gaz de sang montrait une acidose lactique sévère (pH à 7,16, Taux de lactate à 11,2 mmol/l, excès de base à -15,3 mmol/l). L'étude du liquide céphalo-rachidien (LCR) trouvait 200 éléments/mm^3^ à prédominance lymphocytaire. L'échocardiographie montrait une dysfonction myocardique gauche sévère avec une contractilité effondrée et une fraction d'éjection à 36%. L'électrocardiogramme décelait des troubles de repolarisation au niveau des dérivations postérieures gauches. La BNP (Brain Natriuretic Peptide) et les troponines étaient élevées. La PCR (Polymerase Chain Reaction) au niveau sanguin et LCR (Liquide Céphalorachidien) détectait un entérovirus. Le traitement consistait en une semaine de ventilation mécanique associée à la dobutamine et le furosémide. les immunoglobulines n'avaient pas amélioré l'état de notre patient. L'évolution après une semaine d'assistance respiratoire et circulatoire a été marquée par une légère amélioration clinique mais avec une persistance de l'hypocontractilité du ventricule gauche. Le relais per os était fait par le spironolactone, le captopril, la digoxine et l'hydrocortisone. A 12 jours de la non récupération, on introduisait un bolus de corticoïde associé au karvédilol ce qui aurait permis une amélioration progressive. A 1 mois d'hospitalisation, l'amélioration clinique et hémodynamique autorisait la sortie avec comme traitement le karvédilol pendant 2 mois et le captopril pendant 3 mois. Le suivi à l'âge de 5 ans trouvait un enfant en bonne santé est sans aucun médicament.

## Discussion

L'infection néonatale invasive à EV reste rare mais elle peut être rapidement fatale [1, 2]. Le mode principal de transmission est materno-foetal [2]. La symptomatologie est variable allant d'une simple virose à une infection invasive avec défaillance multiviscérale fatale en passant par une septicémie, une méningo-encéphalite, une hépatite, une myocardite ou une coagulopathie intravasculaire disséminée (CIVD) [2, 3]. Certains facteurs de gravité ont été décrits notamment le début avant 7 jours de vie, la prématurité et l'antécédent maternels d'infection à EV [2]. En raison de son large spectre clinique, le diagnostic clinique est souvent trompeur et devrait être suspectée chez tous nouveau né septique. La certitude virologique est faite par la PCR (Polymerase Chain Reaction) qui est plus sensible, plus spécifique et plus rapide que la culture et la sérologie virale [2, 4]. La combinaison de 2 PCR au niveau du sang et du liquide céphalo-rachidien (LCR) même en absence de pléiocytose rachidienne améliore le rendement diagnostique [2, 4].

Devant une suspicion de myocardite, l'évaluation initiale devrait inclure un électrocardiogramme, une échocardiographie et éventuellement une imagerie par résonance magnétique IRM cardiaque qui est en train de devenir un outil important pour le diagnostic et le suivi [3, 5, 6]. Certains facteurs de pronostic sévère ont été décrits notamment la présence d'une myocardite ou une CIVD [1]. La mortalité en cas de myocardite à EV varie entre 44 et 100% et la survie sans séquelles est estimée à 15% [1, 2]. La pathogénie de la myocardite à EV comprend à une nécrose myocardique et une réaction auto-immune [6]. De nouvelles options thérapeutiques immunosuppressives et antivirales spécifiques peuvent améliorer le pronostic mais leur efficacité clinique n'est pas encore clairement démontrée. Des essais cliniques concernant l'interféron bêta, les anticorps monoclonaux et le pléconaril sont en cours [6, 7]. Le traitement de la myocardite aiguë est encore principalement basé sur l'assistance respiratoire et circulatoire [2, 6]. L'Oxygénation par Membrane Extracorporelle (ECMO) devrait être envisagée en cas de myocardite réfractaire [8]. Le traitement de l'insuffisance cardiaque associe des inotropes, des diurétiques et des vasodilatateurs [6]. Les corticoïdes diminueraient la sévérité des myocardites virales; c'est le cas de notre patient. Les données actuelles ne trouvent pas de différence significative dans l'effet du traitement immunosuppresseur des myocardites aiguës probablement en raison du faible effectif [9]. Quant aux immunoglobulines, il n'y a pas suffisamment de données provenant d'études méthodologiquement solides pour recommander leur utilisation systématique, mais elles peuvent être essayées en cas myocardite sévère [10]. En attente d'un traitement spécifique, les mesures de prévention en renforçant les règles d'hygiène dans l'entourage des patients à risques sont évidemment recommandées.

## Conclusion

La myocardite virale, bien que rare, est souvent fatale et devrait être prise dans le diagnostic différentiel chez les nouveaux nés fébrile. L'échocardiographie est un examen capital dans cette démarche et le diagnostic virologique peut être fait rapidement par une PCR. La prise en charge précoce et les mesures de prévention sont les seuls garants d'une diminution de la morbidité et la mortalité qui est considérable.
